# Benzoylaconine suppresses colorectal cancer progression by modulating the CDK2/MMP2 signaling axis

**DOI:** 10.3389/fphar.2026.1902707

**Published:** 2026-10-16

**Authors:** Fengyi Li, Ke Zhang, Miao Wang, Liya Su, Ling Yang

**Affiliations:** 1 Key Laboratory of Medical Cell Biology in Inner Mongolia, Clinical Medicine Research Center, The Affiliated Hospital of Inner Mongolia Medical University, Hohhot, Inner Mongolia, China; 2 First School of Clinical Medicine, Inner Mongolia Medical University, Hohhot, Inner Mongolia, China

**Keywords:** benzoylaconine, cell cycle, colorectal cancer, colorectal cancer organoids, cyclin-dependent kinase 2, matrix metalloproteinase-2, network pharmacology

## Abstract

**Background:**

Colorectal cancer (CRC) has a high incidence, and both metastasis and drug resistance remain formidable challenges in clinical management. Owing to their multi-target activity and favorable safety profiles, natural products have become a prolific source for anticancer drug discovery. Benzoylaconine (BAC) is a secondary metabolite of aconitine with markedly attenuated toxicity that retains anti-inflammatory, analgesic, and antitumor properties; however, its antitumor effects and the underlying molecular mechanisms in CRC are poorly defined. This study was designed to systematically characterize the impact of BAC on the malignant phenotypes of CRC cells and to identify its potential molecular targets.

**Methods:**

HCT116 and SW480 cells were used as model systems. Proliferation was assessed by MTT and EdU assays; migration and invasion by wound-healing and Transwell assays, respectively; cell cycle and apoptosis by flow cytometry. Network pharmacology was applied to predict core targets and construct a protein–protein interaction network. Molecular docking and dynamics simulations examined binding; SPR verified direct binding to CDK2 and MMP2. RNA-seq identified differentially expressed genes and enriched pathways. Western blotting validated key protein changes. Patient-derived CRC organoids (PDOs) were established to evaluate BAC efficacy; organoid viability was assessed by CellTiter-Glo® 2.0 assay, and morphology by bright-field microscopy.

**Results:**

BAC dose- and time-dependently inhibited CRC cell proliferation and EdU positivity, slowed wound closure, and reduced Transwell invasion, indicating impaired migration and invasion. Flow cytometric analysis revealed that BAC induced G1/S cell cycle arrest and apoptosis. Network pharmacology identified CDK2 and MMP2 as key targets; molecular dynamics and SPR confirmed stable binding (KD ≈ 3.9 and 4.4 μM). RNA-seq showed enrichment of pathways related to cell cycle regulation and tumor invasion. Western blotting revealed that BAC dose-dependently downregulated CDK2, CDK6, MMP2, Cyclin E1, and Cyclin D1, and upregulated p21. BAC also inhibited the proliferation of colorectal cancer organoids.

**Conclusion:**

BAC induces G1/S cell cycle arrest in CRC cells, likely through modulation of the CDK2/MMP2 signaling axis, thereby suppressing proliferation, migration, and invasion while promoting apoptosis, and may represent a promising lead for CRC therapy.

## 1 Introduction

Colorectal cancer (CRC) ranks as the third most common malignancy and the second leading cause of cancer-related death worldwide. According to global cancer statistics, over 1.9 million new cases and nearly 1 million deaths are reported annually (Shen et al., 2026; Yu and Xu, 2026). Despite continued advances in early detection, surgical techniques, adjuvant chemotherapy (e.g., FOLFOX and FOLFIRI), targeted therapy (anti-EGFR and anti-VEGF antibodies), and immunotherapy (PD-1 inhibitors), the five-year survival rate for patients with advanced CRC remains below 20% (Georgoulia et al., 2025). Primary or acquired resistance to existing agents is a major driver of treatment failure and mortality (Yang et al., 2026). Identifying anti-CRC agents that act through novel mechanisms and offer favorable efficacy-toxicity profiles therefore represents a pressing clinical need.

Natural products have long been a prolific source of anticancer drug leads. Numerous plant-derived compounds and their derivatives—including paclitaxel and camptothecin—have entered clinical practice as first-line anticancer agents (Newman and Cragg, 2020). Batri-7 is a classic Mongolian medicinal formula prescribed for enteritis and other gastrointestinal disorders. Our group previously demonstrated that Batri-7 markedly suppressed tumor growth in an AOM/DSS mouse model of colorectal cancer (Sun et al., 2026); however, the active constituents responsible for this effect have not been identified. The principal medicinal material in this formula is derived from Aconitum species, and its major component, aconitine, has been reported to possess antitumor activity. Nevertheless, the severe cardiotoxicity of aconitine greatly limits its direct therapeutic application (Ji et al., 2019; Li et al., 2022; Xiang et al., 2023; Zhao et al., 2024). This limitation prompted us to shift focus to the *in vivo* metabolites of aconitine. Drug metabolism studies indicate that aconitine undergoes stepwise hydrolysis of the C8 acetyl group and the C14 benzoyl group, yielding benzoylaconine (BAC) as the main secondary metabolite (Zhuang and Yao, 2025). Compared with aconitine, BAC shows markedly attenuated toxicity while retaining analgesic, anti-inflammatory, and cardioprotective properties (Li et al., 2022; Xing et al., 2022; Zhao et al., 2024). In addition, BAC promotes mitochondrial biogenesis through activation of the AMPK signaling cascade (Deng et al., 2019). Furthermore, BAC has been shown to suppress ovarian cancer cell growth *in vivo* by inhibiting the PI3K/AKT signaling axis (Li et al., 2025), and its anti-inflammatory effects, mediated through the TNF-α/TRAF2/NF-κB pathway, have been validated in an osteoarthritis model (Wang et al., 2025b). Collectively, these observations suggest that BAC may represent a key active form underlying the pharmacological efficacy of aconitine and the Batri-7 formula, while offering a relatively favorable safety profile. To date, however, whether BAC can directly inhibit colorectal cancer remains unexplored. Building on our prior work with this formula, the present study was designed to characterize the direct effects of BAC on CRC cells and to provide experimental evidence for the “toxicity reduction and efficacy retention” paradigm of aconitine-type alkaloids.

Dysregulation of the cell cycle is a fundamental driver of tumor initiation and progression. Cyclin-dependent kinase 2 (CDK2), a pivotal component of the cell cycle machinery, drives the G1/S transition primarily through its interaction with Cyclin E, thereby triggering DNA replication (Fagundes and Teixeira, 2021; Hanahan, 2022). In various human malignancies, including CRC, CDK2 is frequently overexpressed or aberrantly activated, and its expression levels are closely associated with tumor proliferative activity, aggressiveness, and poor prognosis (García-Reyes et al., 2018; Knudsen et al., 2025); CDK2 is therefore regarded as an attractive therapeutic target. In parallel, tumor invasion and metastasis proceed through a multi-step cascade in which degradation of the extracellular matrix (ECM) represents a critical event that enables tumor cells to breach the basement membrane and invade surrounding tissues. Matrix metalloproteinase 2 (MMP2), a prominent member of the MMP family, specifically degrades type IV collagen—a major structural component of the ECM—thereby facilitating tumor cell migration and dissemination (Okura et al., 2025; Shoari et al., 2025; Xu et al., 2025). A substantial body of evidence indicates that MMP2 expression is markedly elevated in CRC tissues relative to adjacent normal tissues, and its overexpression is positively correlated with depth of invasion, lymph node involvement, distant metastasis, and reduced overall survival (Ibraheem, 2024; Wang et al., 2024; Mozooni et al., 2025). Although the roles of CDK2 and MMP2 in CRC progression are well established, natural small-molecule inhibitors capable of concurrently targeting both nodes are exceedingly rare. Network pharmacology offers distinct advantages for elucidating multi-target mechanisms, while molecular dynamics simulations can predict drug–target interactions with reasonable accuracy. Organoids are three-dimensional cellular complexes formed through the self-assembly of stem cells, recapitulating the structural and functional characteristics of their organ of origin, and have been widely used for disease modeling and drug screening (Deng et al., 2019; Vandana et al., 2023). In this study, we integrated these drug discovery approaches with systematic cell-based functional assays, transcriptomic profiling, Western blot validation,and organoid models to provide both theoretical and experimental support for the development of BAC as a novel anti-CRC therapeutic candidate.

## 2 Materials and methods

### 2.1 Reagents

Benzoylaconine (BAC, Cat. No. A0631) was purchased from Chengdu Must Bio-technology Co., Ltd. 3-(4,5-Dimethylthiazol-2-yl)-2,5-diphenyltetrazolium bromide (MTT, Cat. No. M-8180) was obtained from Beijing Solarbio Science and Technology Co., Ltd. 5-Ethynyl-2′-deoxyuridine (EdU, Cat. No. HY-118411) and the Cell Cycle and Apoptosis Analysis Kit (Cat. No. HY-K1071) were purchased from MedChemExpress LLC (United States). The Annexin V-FITC/PI Apoptosis Detection Kit (Cat. No. KGA1101-50) was sourced from Jiangsu KeyGEN BioTECH Co., Ltd. Transwell chambers (Cat. No. 3413) and Corning® Matrigel® matrix (Cat. No. 354234) were obtained from Corning Inc., (United States). RIPA lysis buffer (Cat. No. CW2333S) and the BCA Protein Assay Kit (Cat. No. CW0014S) were purchased from Beijing ComWin Biotech Co., Ltd. (CWBIO). All primary antibodies were sourced from Proteintech (Wuhan Sanying Biotechnology Co., Ltd.) with the following specifications: anti-CDK2 (1:5000, Cat. No. 10122-1-AP), anti-CDK6 (1:1000, Cat. No. 14052-1-AP), anti-Cyclin E1 (1:500, Cat. No. 11554-1-AP), anti-Cyclin D1 (1:5000, Cat. No. 60186-1-Ig), anti-MMP2 (1:1000, Cat. No. 66366-1-Ig), anti-p21 (1:1000, Cat. No. 10355-1-AP), anti-Ki-67 (1:5000, Cat. No. 27309-1-AP), anti-CK7 (1:1000, Cat. No. 17513-1-AP), anti-E-cadherin (1:5000, Cat. No. 20874-1-AP), CDX2 (1:400, Cat. No. 602431-IgG1), anti-CK20 (1:1000, Cat. No. 17329-1-AP), and anti-GAPDH (1:5000, Cat. No. 60001-1-Ig). HRP-conjugated goat anti-rabbit IgG (Cat. No. ab175773) and goat anti-mouse IgG (Cat. No. ab175783) were purchased from Abcam (UK). Colorectal cancer organoid culture medium (Cat. No. ND-1007-CM) and tissue dissociation solution (Cat. No. NBS5322) were purchased from Nanjing Nuodian Biotech. CellTiter-Glo® 2.0 (Cat. G9242) was sourced from Promega (United States).

### 2.2 Cell culture

The human colorectal cancer cell lines HCT116 and SW480 were obtained from the Wuhan Procell system and Biowing Biotechnology, Shanghai, China. Both lines were cultured in Iscove’s Modified Dulbecco’s Medium (IMDM) supplemented with 10% fetal bovine serum and 1% penicillin-streptomycin and maintained at 37 °C in a humidified atmosphere containing 5% CO_2_. All experiments were performed with cells in the logarithmic growth phase.

### 2.3 MTT assay for cell proliferation

Logarithmically growing HCT116 and SW480 cells were harvested by enzymatic digestion, seeded into 96-well plates at 5 × 10^3^ cells per well (100 µL per well), and left to adhere overnight. The medium was then replaced with fresh medium containing BAC at final concentrations of 0, 12.5, 25, 50, 100, and 200 μmol/L, with each concentration tested in quintuplicate. After incubation for 24 h and 48 h, 20 µL of MTT solution (5 mg/mL) was added to each well, and the plates were incubated for an additional 4 h. The supernatant was carefully aspirated, and 150 µL of DMSO was added per well to dissolve the formazan crystals by gentle shaking for 15 min. The optical density (OD) was measured at 490 nm with a microplate reader. Cell viability was calculated as:

$$\text{Cell viability }\left(\%\right)=\left(\text{OD}\_\text{experimental }–\text{ OD}\_\text{blank}\right)\;/\text{×}\;\left(\text{OD}\_\text{control }–\text{ OD}\_\text{blank}\right)\times 100\%.$$

### 2.4 EdU incorporation assay

HCT116 and SW480 cells were seeded in 96-well plates at 5 × 10^3^ cells/well and cultured overnight to permit attachment. The cells were treated with BAC at 0, 50, and 100 μmol/L for 24 h. The EdU incorporation assay was carried out using the EdU Cell Proliferation Detection Kit according to the manufacturer’s protocol. Briefly, EdU working solution (final concentration 10 μmol/L) was added, and the cells were incubated for 4 h. Cells were then fixed with 4% paraformaldehyde for 15 min, permeabilized with 0.5% Triton X-100 for 20 min, and incubated with Click-iT reaction cocktail for 30 min at room temperature in the dark. Nuclei were counterstained with Hoechst 33342. Random images were captured under a fluorescence microscope, and the percentage of EdU-positive cells (green) among Hoechst-positive cells (blue) was quantified using ImageJ.

### 2.5 Colony formation assay

HCT116 and SW480 cells were seeded into 6-well plates at 500 cells per well and allowed to adhere overnight. The cells were then treated with BAC at 0, 50, and 100 μmol/L and cultured for 14 days, with the drug-containing medium replaced every 3 days. Once discrete, visible colonies had formed, the culture was terminated. Colonies were fixed with 4% paraformaldehyde, stained with 0.1% crystal violet, rinsed gently under running water until the background was clear, and air-dried. Images were captured, and the colony formation rate was calculated.

### 2.6 Wound-healing assay

HCT116 and SW480 cells were plated in 6-well plates at 5 × 10^5^ cells per well and grown to approximately 90% confluence. Three parallel scratches were made across the monolayer using a 200 µL sterile pipette tip. Detached cells were removed by gentle washing with PBS three times, after which serum-free medium containing BAC at 0, 50, and 100 μmol/L was added. The same wound fields were photographed at 0 h and 24 h. Wound areas were quantified with ImageJ, and the wound closure rate was calculated as:

$$\text{Wound closure }\left(\%\right)=\left[\left(\text{wound area at }0\;h-\text{wound area at }24\;h\right)/\text{wound area at }0\;h\right]\times 100\%.$$

### 2.7 Transwell invasion assay

Matrigel matrix was diluted 1:8 with serum-free medium, and 50 µL of the dilution was evenly applied to the upper surface of each Transwell insert. The coated inserts were incubated at 37 °C for 1 h to allow gelation. After removal of residual liquid, the membranes were rehydrated with serum-free medium. HCT116 and SW480 cells in logarithmic growth were resuspended in serum-free medium and seeded into the upper chambers at 5 × 10^4^ cells per well. After overnight attachment, the upper-chamber medium was replaced with serum-free medium containing BAC at 0, 50, or 100 μmol/L, and the lower chambers were filled with 600 µL of complete medium containing 20% FBS as a chemoattractant. Following 24 h of incubation, non-invading cells on the upper membrane surface were removed with a cotton swab. Cells that had invaded to the lower surface were fixed with 4% paraformaldehyde, stained with 0.1% crystal violet, and imaged under an inverted microscope. Invaded cells were counted in randomly selected fields.

### 2.8 Cell cycle analysis

HCT116 and SW480 cells were treated with BAC at 0, 50, and 100 μmol/L for 24 h, harvested, washed twice with ice-cold PBS, and fixed in 70% ethanol at 4 °C overnight. The fixed cells were collected by centrifugation, washed with PBS, and resuspended in staining solution containing 50 μg/mL propidium iodide (PI) and 100 μg/mL RNase A. After incubation in the dark at room temperature for 30 min, cells were analyzed by flow cytometry. Red fluorescence was detected with 488 nm excitation, and DNA content histograms were used to determine cell cycle distribution.

### 2.9 Apoptosis assay

Apoptosis was assessed by Annexin V-FITC/PI dual staining. Cells treated with BAC at 0, 50, and 100 μmol/L for 24 h were collected, including floating cells in the supernatant. Following two washes with ice-cold PBS, the cells were resuspended in 500 µL of binding buffer. Subsequently, 5 µL of Annexin V-FITC and 5 µL of PI were added sequentially, mixed gently, and incubated for 10 min at room temperature in the dark. Flow cytometric analysis was performed within 1 h.

### 2.10 Network pharmacology analysis

#### 2.10.1 Prediction of potential BAC targets

The SMILES representation of BAC was obtained from the PubChem database and submitted to the SwissTargetPrediction platform for target prediction, applying a probability threshold of >0.3. All predicted target proteins were mapped to gene symbols using the UniProt database, yielding a set of potential targets for BAC.

#### 2.10.2 Collection of CRC-Associated disease targets

The GeneCards, OMIM, and DisGeNET databases were comprehensively searched using the terms “Colorectal Cancer,” “colon cancer,” and “rectal cancer.” From the GeneCards results, targets with relevance scores exceeding the median were retained and combined with those retrieved from OMIM and DisGeNET. After removal of duplicate entries, a CRC disease-related target set was compiled.

#### 2.10.3 Identification of common targets and construction of PPI network

The intersection of the BAC potential target set and the CRC disease target set was determined using the Venny 2.1 online tool to identify candidate targets of BAC against CRC. These common targets were submitted to the STRING database, with the species restricted to *Homo sapiens* and a minimum interaction confidence threshold of 0.4, to construct a protein–protein interaction (PPI) network. The network was imported into Cytoscape 3.9.1 for visualization, and topological parameters—including degree and betweenness centrality—were computed using the built-in NetworkAnalyzer to identify core targets.

#### 2.10.4 GO and KEGG enrichment analysis

The common targets were uploaded to the DAVID database for Gene Ontology (GO) functional annotation and Kyoto Encyclopedia of Genes and Genomes (KEGG) pathway enrichment analysis, with a significance threshold of P < 0.05. The top 10 enriched GO terms within the molecular function (MF), cellular component (CC), and biological process (BP) categories, along with the top 10 significantly enriched KEGG pathways, were selected for further analysis. Enrichment results were visualized using the ggplot2 package in R.

### 2.11 Molecular docking, molecular dynamics simulation and surface plasmon resonance (SPR) assay

#### 2.11.1 Molecular docking

The crystal structures of CDK2 (PDB ID: 8VQ3) and MMP2 (PDB ID: 7XJO) were obtained from the RCSB Protein Data Bank. Protein structures were prepared with PyMOL by removing water molecules and co-crystallized ligands and adding hydrogen atoms. The 3D structure of BAC was retrieved from PubChem in SDF format and converted to PDB format using OpenBabel. Semi-flexible docking was performed with AutoDock Vina 1.1.2. The grid box was centered on the original ligand-binding site, and its dimensions were set to enclose the entire binding pocket. Binding affinity was evaluated from the docking score (kcal/mol), and the best-docked poses were visualized in PyMOL.

#### 2.11.2 Molecular dynamics simulation

Molecular dynamics (MD) simulations were carried out using GROMACS 2022.2. Force field parameters for the protein were generated with the pdb2gmx tool. For the ligand, GAFF2-compatible topology files were built using Sobtop 1.0 (dev3.1), and partial charges were assigned by the RESP method to ensure physically meaningful charge distributions. The Amber14SB force field was applied to the receptor proteins. Each complex was solvated in a cubic TIP3P water box with a 1 nm buffer distance, and counterions were added to neutralize the system. Simulations were run under NPT conditions at 310 K and 1 bar; temperature was controlled with the Nosé–Hoover thermostat and pressure with the Parrinello–Rahman barostat. A 100 ns production run was performed using a 2 fs integration time step. Root-mean-square deviation (RMSD), root-mean-square fluctuation (RMSF), hydrogen bond count, radius of gyration (Rg), solvent-accessible surface area (SASA), and the free energy landscape were computed to assess complex stability, structural fluctuations, and solvation effects.

#### 2.11.3 Surface plasmon resonance (SPR) assay

The binding affinity of benzoylaconine for MMP2 and CDK2 was determined at 25 °C using a Biacore 1K system (Cytiva, Sweden). MMP2 and CDK2 were immobilized on a Series S Sensor Chip CM5 via amine coupling. Briefly, the chip surface was activated with an NHS/EDC mixture. Proteins were diluted to 40 μg/mL in sodium acetate buffer (pH 4.0) for immobilization, and residual active sites were subsequently blocked with ethanolamine-HCl. Immobilization levels of approximately 8000 RU for MMP2 and 3000 RU for CDK2 were achieved. PBST was used as the running buffer at a flow rate of 30 μL/min. Serially diluted benzoylaconine samples were injected with a 90-s association phase and a 120-s dissociation phase. The chip surface was regenerated with glycine buffer (pH 3.0). Data were acquired and analyzed using Biacore Insight Evaluation Software. Kinetic parameters—association rate constant (k_a_), dissociation rate constant (k_d_), and equilibrium dissociation constant K_D_ = k_d_/k_a_ were obtained by fitting to a 1:1 binding model. The steady-state affinity model was also used as a supplementary fitting approach.

### 2.12 RNA sequencing (RNA-seq)

Cells were divided into a control group (0 μmol/L BAC) and a BAC-treated group (100 μmol/L BAC, 24 h treatment of HCT116 cells), with three biological replicates per group. Total RNA was extracted and subjected to transcriptome sequencing on the Illumina platform. Differentially expressed genes (DEGs) were identified using DESeq2 with the criteria |log_2_ fold change| > 1 and adjusted P < 0.05. GO and KEGG enrichment analyses were performed with the clusterProfiler package to characterize the biological processes and pathways altered in CRC cells following BAC treatment.

### 2.13 Western blot analysis

HCT116 and SW480 cells were treated with BAC at 0, 50, and 100 μmol/L for 48 h and then harvested. Total protein was extracted on ice using RIPA lysis buffer supplemented with protease and phosphatase inhibitors and quantified with a BCA protein assay kit. Equal amounts of protein (30 µg) were separated by SDS-PAGE and transferred to nitrocellulose membranes. Membranes were blocked with 5% non-fat milk for 1 h at room temperature and then incubated overnight at 4 °C with individual primary antibodies against CDK2 (1:5,000), MMP2 (1:1,000), Cyclin E1 (1:500),CDK6 (1:1,000),Cyclin D1 (1:5,000), p21 (1:1,000), and GAPDH (1:5,000), applied separately. After three washes with TBST, the membranes were incubated with HRP-conjugated secondary antibodies (1:10,000) for 1 h at room temperature in the dark. Following three additional washes, protein bands were visualized with a fluorescence imaging system. For proteins of similar molecular weight, the same membrane was incubated with membrane stripping buffer to remove bound antibodies and subsequently reprobed with other primary antibodies. Band intensities were quantified using ImageJ, and the relative expression of each target protein was normalized to GAPDH.

### 2.14 Patient-derived colorectal cancer organoid culture and drug treatment

This study was approved by the local ethics committee (approval no. KY.2021044). Fresh colorectal cancer tissues were collected from surgical specimens, minced, and digested with a commercially available tissue dissociation solution. The resulting cell clusters were filtered through a 100-µm cell strainer, resuspended in Matrigel, and seeded into 24-well plates at 50 µL per well. Organoids were cultured in colorectal cancer organoid medium. The culture medium was replaced every 3 days, and organoids were passaged every 7–10 days. To verify phenotypic concordance with the original tumors, paraffin sections of patient-derived tissues and matched organoids were subjected to H&E staining and immunohistochemical analysis for Ki-67, CK7, CK20, CDX2, and E-cadherin. For drug testing, colorectal cancer organoids were seeded into 96-well plates with 5 µL of Matrigel per well and treated with BAC at concentrations of 0, 25, 50, 100, 200, 400, and 800 μmol/L for 6 days. Morphological changes were assessed by bright-field imaging. Cell viability was measured using the CellTiter-Glo® 2.0 reagent, and IC50 values were calculated by nonlinear regression using GraphPad Prism 9.0.

### 2.15 Statistical analysis

Statistical analyses were conducted utilizing GraphPad Prism 9.0. Each *in vitro* experiment was independently replicated a minimum of three times. Data are expressed as mean ± standard deviation. For comparisons between two groups, either Student’s t-test or the Mann-Whitney U test was employed, depending on the data distribution. Comparisons among multiple groups were conducted using one-way ANOVA, followed by appropriate *post hoc* tests. Correlation analyses were performed using either Pearson or Spearman correlation methods. A p-value of less than 0.05 was considered indicative of statistical significance.

## 3 Results

### 3.1 BAC suppresses the proliferation, migration, and invasion of CRC cells

The chemical structure of benzoylaconine (BAC) is shown in Figure 1A. BAC inhibited the proliferation of HCT116 and SW480 cells in a dose- and time-dependent manner (Figure 1B), with 24-h IC_50_ values of 86 μmol/L and 83.28 μmol/L, respectively. Based on these values, 50 and 100 μmol/L were chosen for subsequent *in vitro* assays. EdU incorporation demonstrated that BAC markedly reduced the proportion of EdU-positive cells (Figure 1C), and the colony formation assay confirmed a concentration-dependent decline in clonogenic capacity (Figure 1D). Transwell invasion experiments and Wound-healing assays further revealed that BAC impaired the migratory and invasive abilities of CRC cells in a dose-dependent fashion (Figures 1E,F).

**FIGURE 1:**
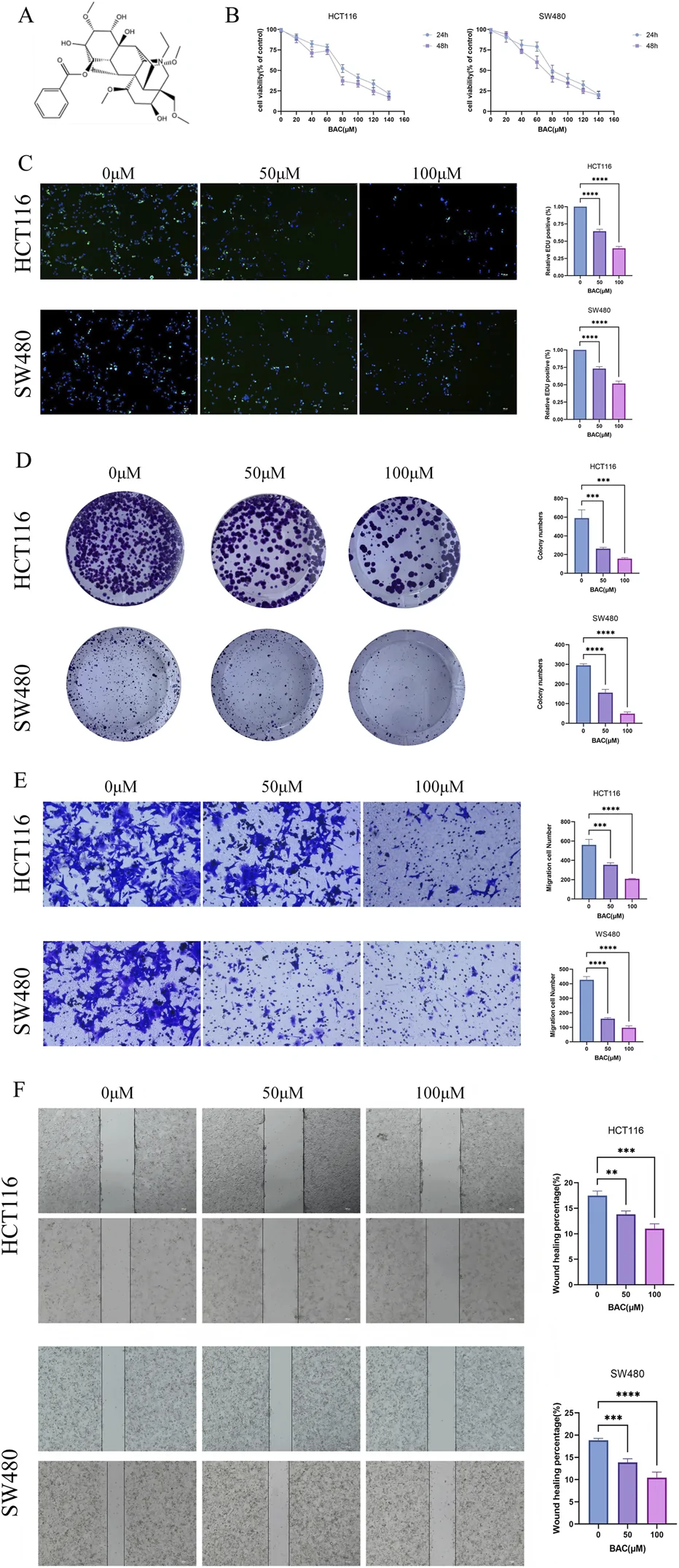
Benzoylaconine (BAC) suppresses malignant phenotypes of CRC HCT116 and SW480 cells in a dose- and time-dependent manner. **(A)** Chemical structure of BAC. **(B)** MTT assay measuring cell viability of HCT116 and SW480 cells treated with increasing concentrations of BAC (0, 12.5, 25, 50, 100, 200 μmol/L) for 24 and 48 h. The IC50 values at 24 h were 86 μmol/L for HCT116 and 83.28 μmol/L for SW480. **(C)** EdU incorporation assay. Representative images and quantification of EdU-positive proliferating cells after 24 h exposure to 0, 50, and 100 μmol/L BAC. Green, EdU; blue, Hoechst 33342. **(D)** Colony formation assay. Cells were cultured with the indicated concentrations of BAC for 14 days, and colonies were visualized by crystal violet staining and counted. **(E)** Transwell invasion assay. Cells that invaded through the Matrigel-coated membrane were fixed, stained, and counted. **(F)** Wound-healing assay. Horizontal migration was assessed by the percentage of wound closure after 24 h of serum-free BAC treatment. Data are presented as mean ± SD from at least three independent biological replicates. *P < 0.05, **P < 0.01, ***P < 0.001, ****P < 0.0001 compared with the 0 μmol/L control group.

### 3.2 Network pharmacology identifies CDK2 and MMP2 as core targets of BAC in CRC

A network pharmacology approach was employed to predict the candidate targets of BAC against CRC. Following database retrieval and deduplication, 103 BAC-related targets and 1,727 CRC-associated targets were collected, and their intersection yielded 29 potential therapeutic targets (Figure 2A). A protein–protein interaction (PPI) network constructed from these shared targets highlighted evident interactions among CDK2, MMP2, and CCNE1 (Figures 2B,C). GO enrichment analysis indicated that the common targets were predominantly involved in biological processes such as cell cycle progression, protein phosphorylation, and positive regulation of cell migration (Figure 2D). KEGG pathway analysis further showed significant enrichment in inflammation-associated signaling pathways and the cell cycle pathway (Figure 2E). Collectively, these results suggest that BAC may suppress CRC progression by interfering with cell cycle- and migration-related pathways.

**FIGURE 2:**
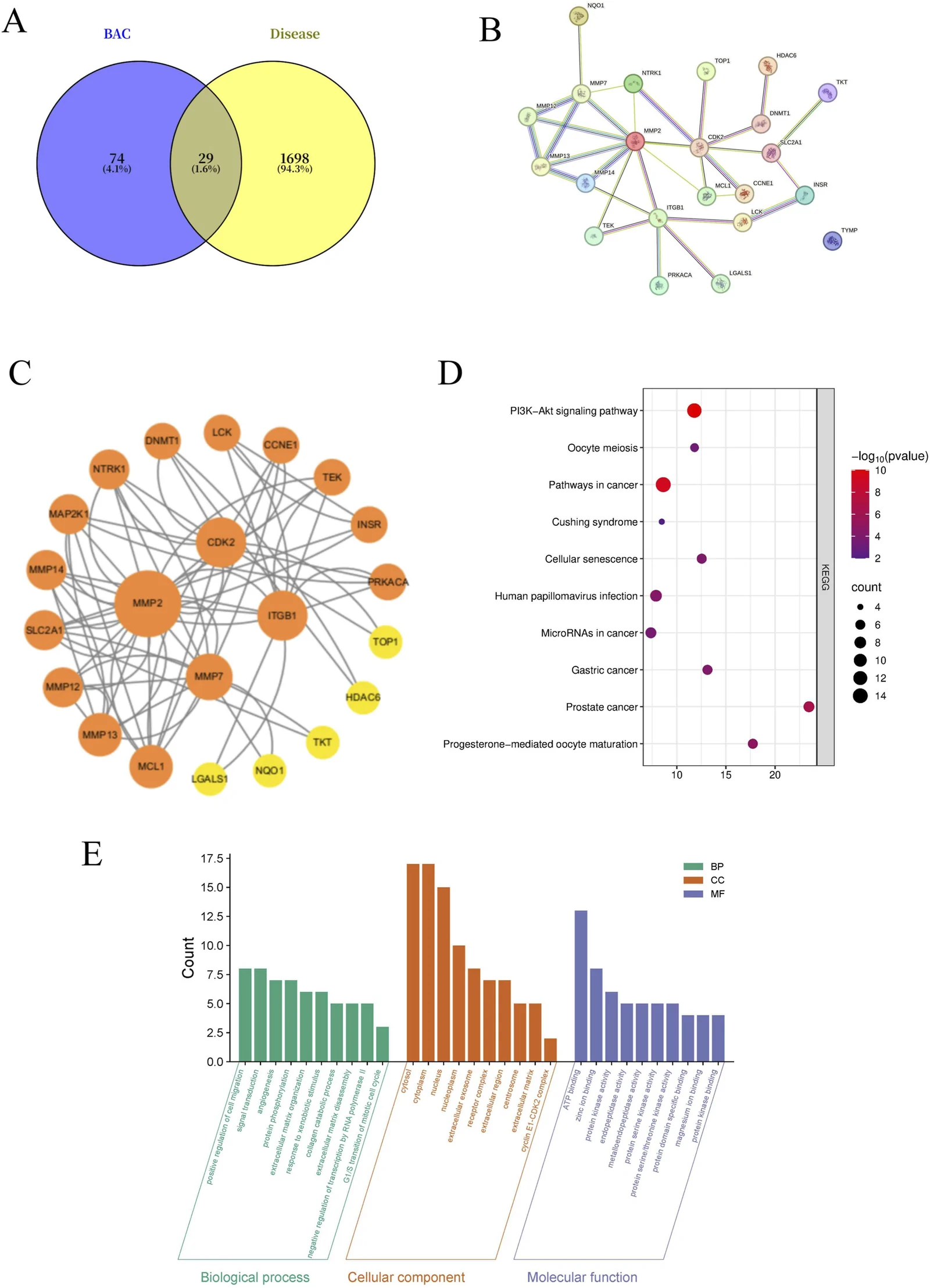
Identification of core therapeutic targets of BAC against CRC and functional enrichment analysis using network pharmacology. **(A)** Venn diagram showing 29 overlapping targets between 103 potential BAC-related targets and 1727 CRC-associated disease targets. **(B)** Protein–protein interaction (PPI) network of the 29 shared targets. Node size is proportional to the topological degree; CDK2 and MMP2 were identified as hub genes. **(C)** Bar chart displaying the topological ranking of core nodes within the PPI network. **(D)** Bubble plot of the top 10 enriched KEGG pathways. Targets were significantly clustered in cell cycle and inflammation-associated signaling pathways. **(E)** Top 10 enriched Gene Ontology (GO) terms across biological process (BP), cellular component (CC), and molecular function (MF). The terms were predominantly associated with cell cycle regulation and cell migration modulation.

### 3.3 Molecular docking, molecular dynamics simulations, and SPR analysis confirm stable binding of BAC to CDK2 and MMP2

To determine whether BAC directly binds the two core targets, molecular docking was first performed. The docking results gave binding free energies of −8.13 kcal/mol for CDK2 and −8.06 kcal/mol for MMP2, indicating favorable binding affinity (Figure 3A). The stability of these binding modes was further evaluated by 100 ns molecular dynamics simulations. The root-mean-square deviation (RMSD) values remained below 0.3 nm throughout the simulations, reflecting stable binding of both the CDK2–BAC and MMP2–BAC complexes (Figure 3B). The radius of gyration (Rg) exhibited negligible fluctuation, supporting the structural compactness of the protein–ligand complexes (Figure 3C). Solvent-accessible surface area (SASA) analysis showed no marked changes after ligand binding, consistent with the formation of stable complexes (Figure 3D). Root-mean-square fluctuation (RMSF) values were predominantly below 0.4 nm for both complexes, indicative of low flexibility and high stability (Figure 3E). Free energy landscape (FEL) plots revealed that the CDK2–BAC complex adopted a low-energy conformation around PC1 ≈ 2.55, PC2 ≈ 0.28, whereas the MMP2–BAC complex displayed its lowest free energy near PC1 ≈ 1.49, PC2 ≈ 0.17, suggesting that these conformations are thermodynamically favored (Figure 3F). SPR results demonstrated stable and concentration-dependent binding signals in both experiments, indicating specific binding. The affinities were calculated by fitting with the “Steady state Affinity Fit Model,” yielding KD values of 3.91 μM for CDK2 and 4.44 μM for MMP2 (Figure 3G).

**FIGURE 3:**
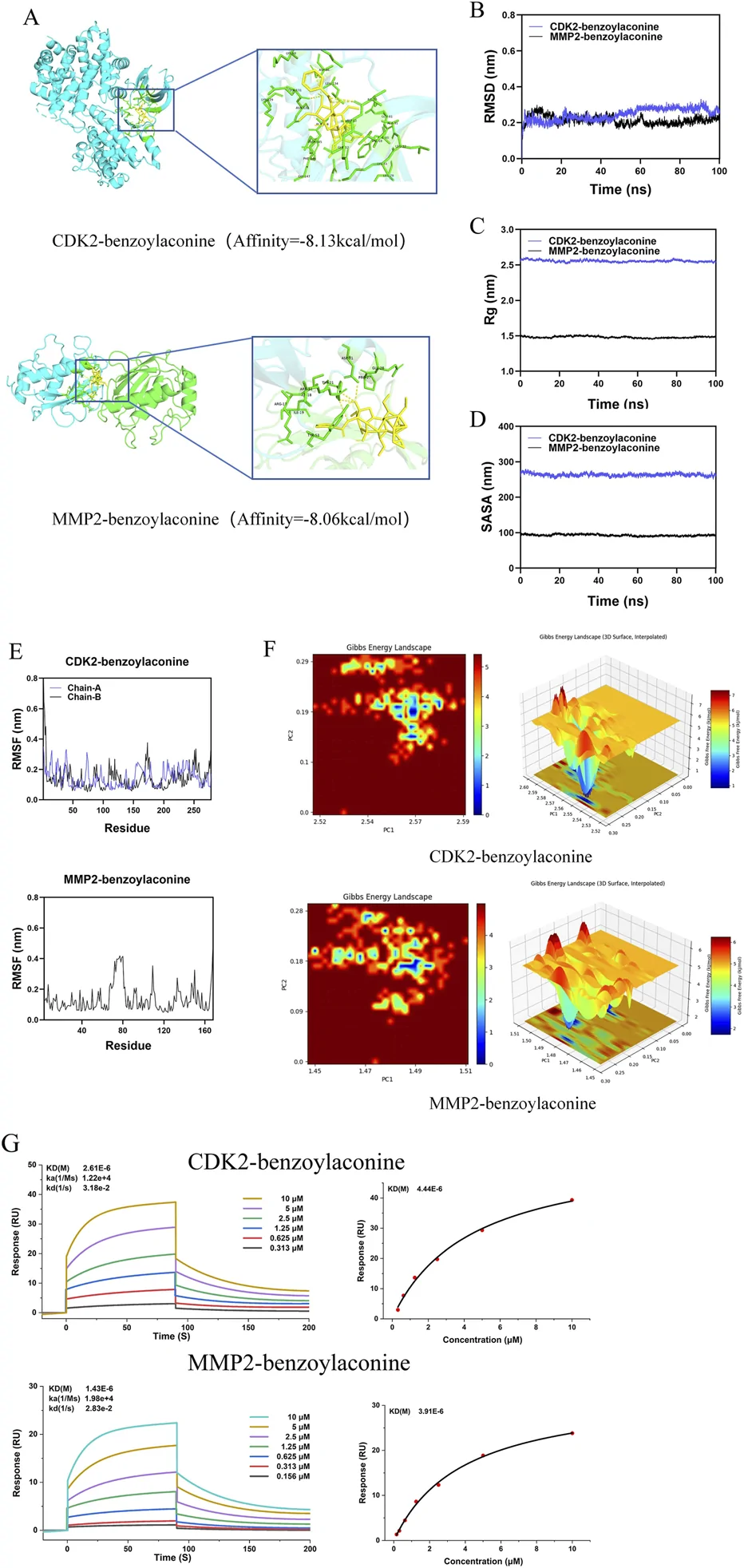
Molecular Docking, Molecular Dynamics Simulations, and SPR Analysis Confirm Stable Binding of BAC to CDK2 and MMP2. **(A)** PyMOL visualization of the docking conformations. BAC binds to the active pockets of CDK2 (PDB ID: 8VQ3) and MMP2 (PDB ID: 7XJO), with binding free energies of −8.13 kcal/mol and −8.06 kcal/mol, respectively. **(B)** Root-mean-square deviation (RMSD) curves of the CDK2–BAC and MMP2–BAC complexes over the 100-ns simulation. RMSD values remained below 0.3 nm, indicating conformational stability. **(C)** Radius of gyration (Rg) curves, showing negligible fluctuations throughout the simulation. **(D)** Solvent-accessible surface area (SASA) curves for the two complexes. **(E)** Root-mean-square fluctuation (RMSF) curves of the protein backbones. Most residues exhibited fluctuations below 0.4 nm. **(F)** Free energy landscape (FEL) plots, revealing thermodynamically favorable low-energy conformations for each complex. **(G) **The SPR results are presented as sensorgrams (surface bound) and fitting plots. The colored curves represent sensorgrams, with different colors indicating different concentrations. The parabolic curve represents the steady-state fitting plot, with different points corresponding to different concentrations.

### 3.4 BAC induces G1/S cell cycle arrest and promotes apoptosis in CRC cells

Given the cell cycle pathway identified by network pharmacology, flow cytometry was used to examine changes in cell cycle distribution. Compared with the control group, treatment with 50 μmol/L and 100 μmol/L BAC for 24 h markedly increased the G1 population and concomitantly reduced the S populations in both HCT116 and SW480 cells, indicating that BAC effectively arrests CRC cells at the G1/S transition (Figure 4A). Annexin V-FITC/PI dual staining further revealed a pronounced, dose-dependent increase in the percentage of apoptotic cells, demonstrating that BAC not only suppresses proliferation but also potently triggers apoptosis (Figure 4B).

**FIGURE 4:**
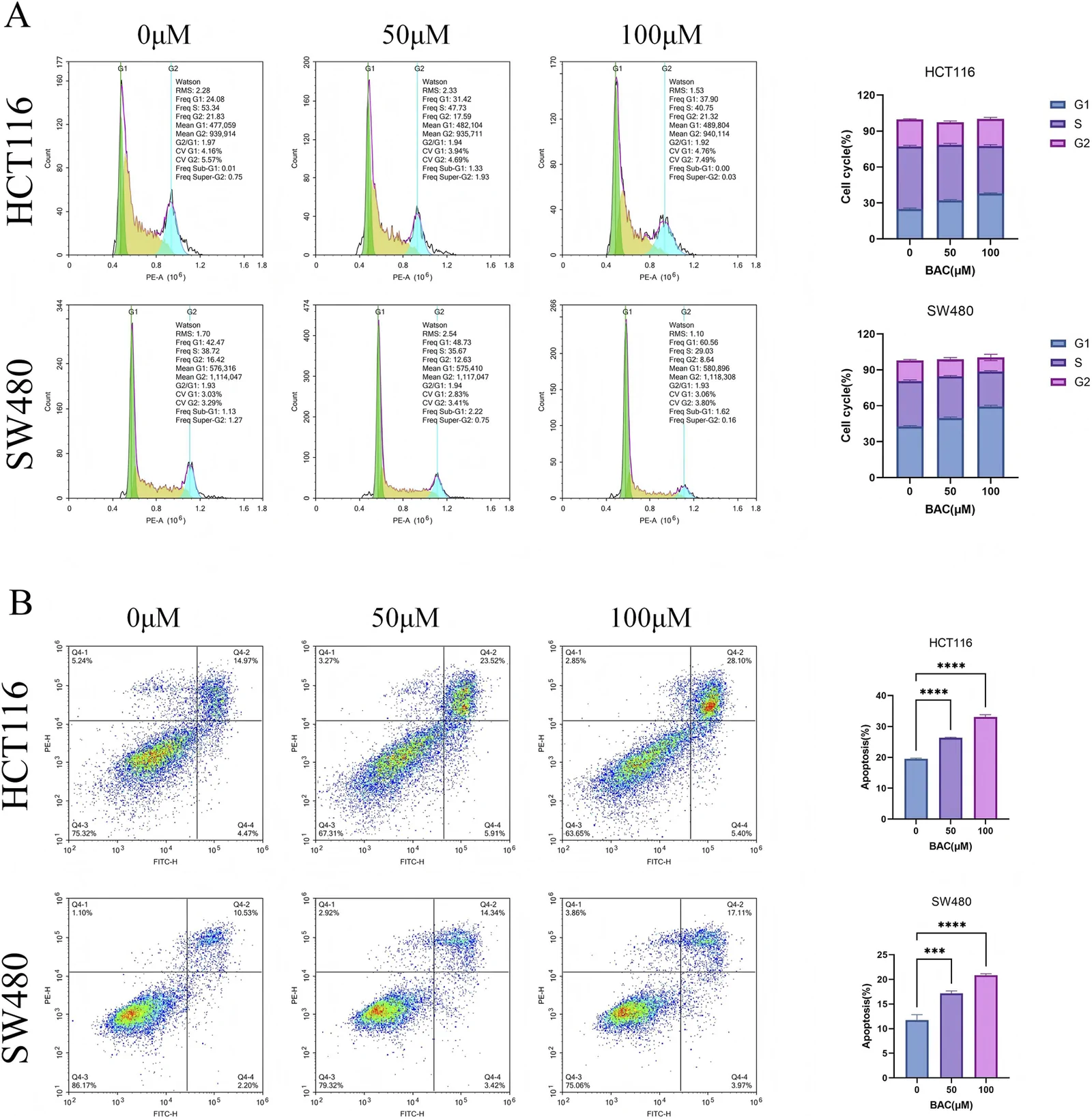
BAC induces G1/S cell cycle arrest and apoptosis in HCT116 and SW480 CRC cells. Cells were treated with 0, 50, and 100 μmol/L BAC for 24 h and then analyzed by flow cytometry. **(A)** Representative histograms of PI-stained cell cycle distribution and quantification. BAC-treated groups exhibited an increased G1 population and decreased S population. **(B)** Representative dot plots of Annexin V-FITC/PI staining and quantification of apoptosis. BAC increased the total apoptotic rate in a dose-dependent manner. Data are presented as mean ± SD. *P < 0.05, **P < 0.01, ***P < 0.001, ****P < 0.0001 compared with the 0 μmol/L control group.

### 3.5 Transcriptome sequencing and western blotting reveal that BAC suppresses the CDK2/MMP2 signaling axis

RNA-seq analysis comparing control and BAC-treated HCT116 cells identified 1111 differentially expressed genes (DEGs), comprising 424 upregulated and 687 downregulated transcripts (Figure 5A). GO enrichment analysis showed that these DEGs were predominantly associated with cell cycle regulation (Figure 5B), and KEGG pathway analysis similarly highlighted enrichment in the cell cycle pathway (Figure 5C). Several key cell cycle regulators—including *CDK2*, *CCNE1*, *CDK6*, *CCND1* and *CDKN1A*—displayed marked transcriptional changes, corroborating the earlier network pharmacology predictions and cell-based functional assays. Validation at the protein level by Western blotting demonstrated a dose-dependent decrease in CDK2 and MMP2 protein levels in both HCT116 and SW480 cells after 48 h treatment with 50 and 100 μmol/L BAC, consistent with the binding predicted by molecular simulations. Cyclin D1 and Cyclin E1 were also downregulated in a concentration-dependent manner, whereas the cyclin-dependent kinase inhibitor p21 was markedly upregulated (Figure 5D). Together with the cell cycle distribution data, these results support a mechanism whereby BAC exerts anti-CRC effects by modulating the CDK2/MMP2 axis, downregulating Cyclin E1/D1, and upregulating p21.

**FIGURE 5:**
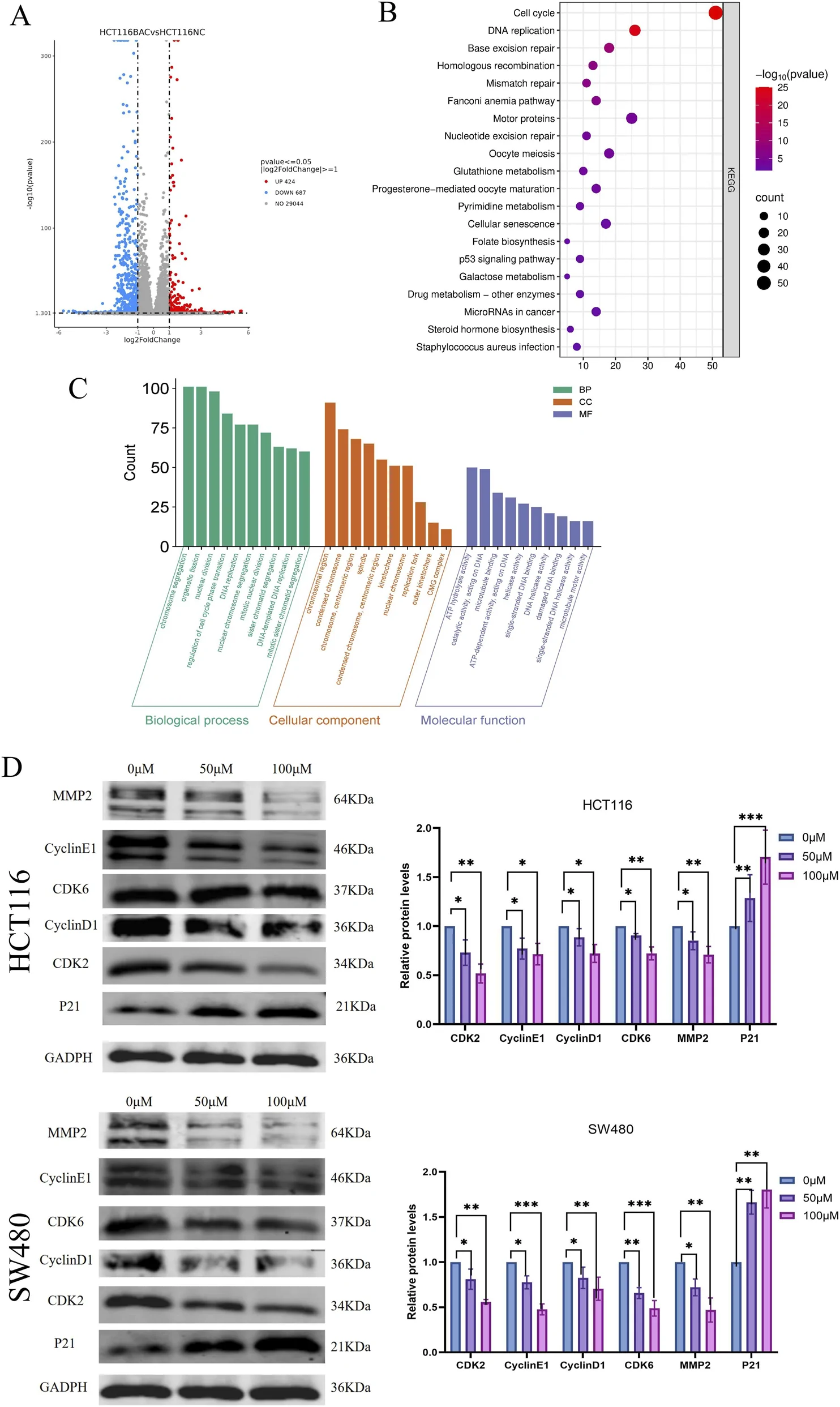
Transcriptome sequencing and Western blotting confirm BAC-mediated regulation of core proteins in the CDK2/MMP2 signaling axis. **(A)** Volcano plot of differentially expressed genes (DEGs) identified by RNA-seq in HCT116 cells treated with 0 and 100 μmol/L BAC for 24 h. DEGs were defined using thresholds of |log2 fold change|>1 and adjusted P < 0.05; 424 genes were upregulated and 687 were downregulated. **(B)** GO enrichment analysis showing that DEGs were predominantly enriched in cell cycle-related biological processes. **(C)** KEGG pathway enrichment analysis indicating that DEGs were clustered in the cell cycle pathway. **(D)** Representative Western blot images and densitometric quantification of core proteins after 48 h of BAC treatment (0, 50, 100 μmol/L). Dose-dependent downregulation of CDK2, MMP2, CDK6, Cyclin D1, and Cyclin E1, and upregulation of p21, were detected in both HCT116 and SW480 cells. GAPDH was used as a loading control. Data are presented as mean ± SD. *P < 0.05, **P < 0.01, ***P < 0.001, ****P < 0.0001 compared with the 0 μmol/L control group.

### 3.6 Phenotypic characterization, and drug sensitivity testing of patient-derived colorectal cancer organoids (PDOs)

To evaluate the activity of BAC in a more clinically relevant model, five patient-derived colorectal cancer organoid models were established. Immunohistochemical analysis confirmed that the established organoids were consistent with the original tumor tissues in the expression of KI67, CK7, E-cadherin, CDX2, and CK20 (Figure 6A). Bright-field imaging showed that BAC treatment led to reduced proliferative activity of colorectal cancer organoids, with morphological changes including reduced size and decreased budding (Figure 6B). The CellTiter-Glo® 2.0 cell viability assay showed that BAC reduced organoid viability, with variable half-maximal inhibitory concentration (IC50) values across the five PDO lines, with IC_50_ values ranging from 300 to 500 μmol/L (Figure 6C).

**FIGURE 6:**
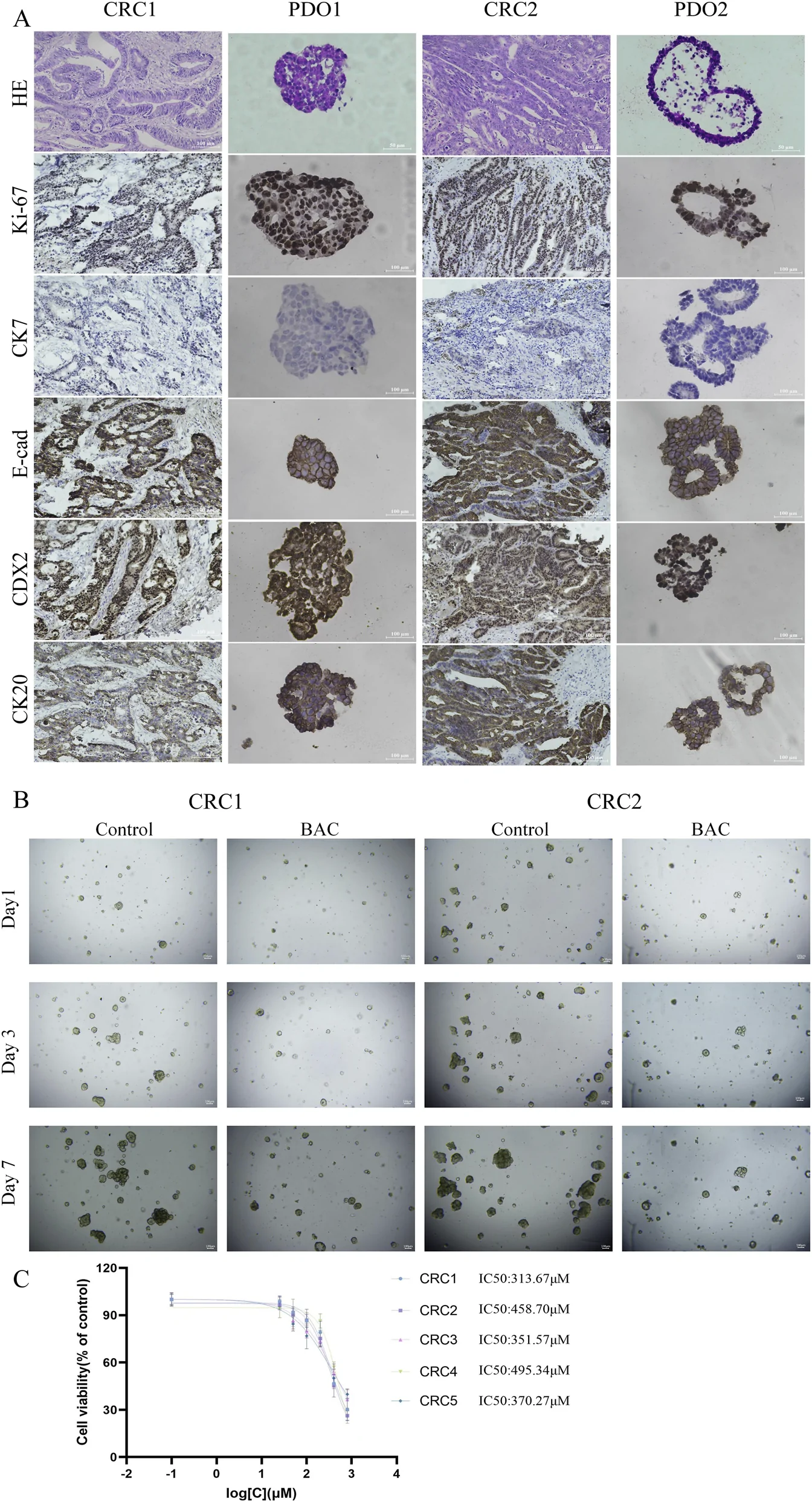
BAC Inhibits Proliferative Activity of Colorectal Cancer Organoids. **(A)** H&E and immunohistochemical staining (Ki-67, CK7, E-cadherin, CDX2, CK20) of primary CRC tissues (CRC1, CRC2) and matched PDOs (PDO1, PDO2). PDOs recapitulate the histological features and marker expression profiles of the corresponding parental tumors. **(B)** Representative bright-field images of untreated control and BAC-treated CRC organoids on days 1, 3, and 7. **(C)** Dose-response viability curves of five PDO lines (CRC1-CRC5) treated with BAC. IC_50_ values for each organoid line are shown. Viability was normalized to the untreated control.

## 4 Discussion

Building on our previous work on the Mongolian medicinal formula Batri-7, this study demonstrates that benzoylaconine (BAC), an *in vivo* metabolite of aconitine from the formula’s principal herb, retains anti-CRC activity with markedly reduced toxicity. These findings provide new experimental evidence supporting the search for anti-CRC agents that act through novel mechanisms and offer favorable efficacy and safety profiles.

The BAC concentrations used for mechanistic studies—50 μmol/L (slightly below the IC_50_) and 100 μmol/L (slightly above the IC_50_)—were selected based on the IC_50_ values obtained for HCT116 cells (approximately 86 μmol/L) and SW480 cells (approximately 83.28 μmol/L). This range was chosen to ensure clear phenotypic effects while avoiding nonspecific cytotoxicity that could arise at excessively high concentrations. The concentrations employed are broadly comparable to the active concentrations of BAC reported in other cell-based models. For instance, Li et al. found that 200 μmol/L BAC did not cause overt toxicity in cardiomyocytes and that BAC inhibited ovarian cancer growth in a mouse model (Li et al., 2025). Ye et al. reported that 30–500 μmol/L BAC only reduced heart rate in zebrafish without producing structural cardiac damage (Ye et al., 2021). Although systematic pharmacokinetic data on *in vivo* exposure levels of BAC as an aconitine metabolite remain limited (Zhuang and Yao, 2025), available evidence indicates that plasma BAC concentrations can reach micromolar to sub-millimolar levels following oral administration of Aconitum herbs (Fan et al., 2021). The concentrations used in this study are therefore of potential biological relevance and are supported by existing literature. Future studies will integrate *in vivo* pharmacokinetic parameters to further optimize dosing regimens.

CDK2 is a key kinase that drives the G1-to-S phase transition (Cornwell et al., 2024; Dragoi et al., 2024). In many tumor types, overexpression or gene amplification of *CDK2* is associated with aggressive growth and poor prognosis (Alzrigat et al., 2026). With growing recognition of the role of CDK2 in drug resistance, the development of novel, highly selective CDK2 inhibitors has regained momentum in anticancer drug discovery (Luo et al., 2026). Our molecular dynamics simulations indicated that BAC can stably occupy the ATP-binding pocket of CDK2, and Western blot analysis further confirmed that BAC downregulates CDK2 protein levels, suggesting that BAC may act as an ATP-competitive inhibitor or affect CDK2 protein stability. More importantly, BAC markedly upregulated the endogenous CDK2 inhibitor p21 while concurrently reducing the expression of the upstream activators Cyclin E1 and Cyclin D1. Thus, BAC appears to inhibit CDK2 activity through three coordinated actions: direct binding, attenuation of activating signals, and enhancement of endogenous inhibition. However, whether BAC directly inhibits CDK2 kinase activity remains to be confirmed by *in vitro* enzymatic assays; the present data support modulation of the CDK2 pathway through reduced CDK2 protein levels and enhanced p21 expression. This multi-layered regulatory mechanism may account for the efficient G1 arrest induced by BAC and could potentially lower the risk of developing drug resistance.

Matrix metalloproteinase 2 (MMP2) plays a critical role in tumor invasion and metastasis by degrading type IV collagen in the basement membrane and extracellular matrix, thereby facilitating tumor cell dissemination. Extensive clinicopathological studies have demonstrated that elevated MMP2 expression in CRC tissues is closely associated with lymph node metastasis, liver metastasis, and shortened overall survival (Ibraheem, 2024; Wang et al., 2024; Xiao et al., 2025). Targeting MMP2 activity or expression therefore represents a promising anti-metastatic strategy. This study revealed that BAC markedly suppressed MMP2 expression in CRC cells, an effect that aligns closely with its ability to inhibit cell migration and invasion. These findings suggest that, in addition to directly inhibiting tumor cell proliferation, BAC may also reshape the tumor microenvironment by reducing extracellular matrix degradation, thereby attenuating the metastatic potential of CRC.

A comprehensive review of the literature suggests that cyclin-dependent kinases (CDKs) and matrix metalloproteinases (MMPs) are not functionally independent and that potential crosstalk may exist between these protein families. For instance, CDK6 has been reported to facilitate the recruitment of the transcription factors c-Jun and Sp1 to the MMP2 promoter (Luo et al., 2020). BAC concurrently modulates CDK2 and MMP2, and may therefore not only exert independent inhibitory effects on proliferation and metastasis but also cooperatively disrupt the malignant crosstalk between these two critical pathogenic nodes. This hypothesis is supported by both network pharmacology analysis and RNA-seq data, which further revealed marked alterations in downstream signaling pathways associated with these targets.

The use of patient-derived organoids (PDOs) further enhances the translational relevance of the findings. Unlike immortalized cell lines, PDOs recapitulate the histopathological and molecular heterogeneity of the original tumors, as confirmed by immunohistochemical staining. We found that the IC_50_ values of BAC in five CRC-PDO lines were notably higher than those obtained from 2D HCT116 and SW480 cell lines. Such potency discrepancy between monolayer cells and 3D organoids is commonly observed for natural-derived small-molecule agents. Several contributing factors should be considered: the Matrigel matrix and compact 3D glandular architecture impede drug diffusion into inner organoid layers; inter-patient tumor heterogeneity leads to variable drug responses among individual PDO lines; and a subset of quiescent non-proliferative cells within organoid spheroids exhibit reduced susceptibility to this cell-cycle-targeting compound. Despite elevated IC_50_ values, bright-field microscopy still revealed obvious morphological deterioration, shrinkage and defective budding in BAC-treated PDOs, confirming the anti-proliferative activity of BAC in this clinically-relevant model. Of note, Matrigel-embedded tumor-only PDOs lack stromal, immune compartments and vascular perfusion found in native tumors, which may further alter drug efficacy *in vivo*. Collectively, our PDO results support the preclinical anti-CRC potential of BAC and highlight that 2D monolayer assays tend to overestimate drug potency; 3D PDO models therefore serve as a more rigorous pre-clinical platform prior to *in vivo* studies. These *in vitro* data provide a more clinically relevant context for evaluating the efficacy of BAC.

Although this study demonstrates the anti-CRC activity of BAC, several limitations warrant further investigation. First, this work was primarily conducted at the *in vitro* level, and *in vivo* efficacy validation in whole-animal models is absent. The absorption, distribution, metabolism, and excretion (ADME) properties of BAC remain poorly defined, and it is uncertain whether effective exposure concentrations in solid tumor tissues can reach levels comparable to those used *in vitro*. Second, the lack of a comprehensive toxicological safety evaluation constitutes another important shortcoming. Although the acute toxicity of BAC is markedly reduced as a detoxified metabolite of aconitine, systematic safety assessment has not been performed. Cardiac safety is an indispensable consideration for Aconitum-derived compounds, and establishing a relatively safe therapeutic window will directly determine the developmental prospects of BAC as an anti-CRC candidate. Future studies should therefore employ subcutaneous xenograft and tail-vein injection lung metastasis models in nude mice to systematically evaluate the *in vivo* antitumor efficacy and pharmacokinetic characteristics of BAC. Third, although network pharmacology and RNA-seq analyses have indicated alterations in multiple signaling pathways, the precise transcriptional regulatory networks downstream of CDK2/MMP2 require deeper elucidation to fully delineate the molecular landscape of BAC-mediated CRC suppression. The anti-CRC effects of BAC may extend beyond the CDK2/MMP2 axis, potentially involving additional pathways such as inflammation-associated and PI3K-AKT signaling. Given existing literature, it remains to be explored whether BAC also regulates the CDK2-Rb pathway, maintaining Rb in a hypophosphorylated state and thereby restricting E2F transcriptional activity to enforce G1 arrest. Finally, whether BAC exerts synergistic or toxicity-reducing effects in combination with established anti-CRC agents such as irinotecan and regorafenib represents a clinically relevant direction that merits systematic investigation.

## Data Availability

The data presented in the study are deposited in the Genome Sequence Archive repository, accession number HRA020857.
